# Association between central obesity and incident diabetes mellitus among Japanese: a retrospective cohort study using propensity score matching

**DOI:** 10.1038/s41598-022-17837-1

**Published:** 2022-08-04

**Authors:** Changchun Cao, Haofei Hu, Xiaodan Zheng, Xiaohua Zhang, Yulong Wang, Yongcheng He

**Affiliations:** 1 Department of Rehabilitation, Shenzhen Dapeng New District Nan’ao People’s Hospital, No. 6, Renmin Road, Dapeng New District, Shenzhen, 518000 Guangdong China; 2 Department of Nephrology, The First Affiliated Hospital of Shenzhen University, Shenzhen, 518000 Guangdong China; 3 Department of Neurology, Peking University Shenzhen Hospital, Shenzhen, 518000 Guangdong China; 4 Department of Nephrology, Shenzhen Hengsheng Hospital, No. 20 Yintian Road, Xixiang Street, Baoan District, Shenzhen, 518000 Guangdong China; 5 Department of Nephrology, Affiliated Hospital of North Sichuan Medical College, No. 1 Maoyuan South Road, Nanchong, 637000 Sichuan China

**Keywords:** Diabetes, Obesity

## Abstract

Previous evidence revealed that central obesity played a vital role in the development of diabetes mellitus (DM). However, because of imbalanced confounding variables, some studies have not wholly established the association between central obesity and diabetes. Propensity score matching (PSM) analysis can minimize the impact of potential confounding variables. Therefore, the aim of the present study was to explore the relationship between central obesity and diabetes in the Japanese population by using PSM analysis. This retrospective cohort study included 15,453 Japanese adults who were free of diabetes at baseline between 2004 and 2015, which provided all medical records for individuals participating in the physical exam. Central obesity at baseline was an independent variable, and incident diabetes during follow-up was an outcome variable. Using a 1:1 PSM analysis, the present retrospective cohort study included 1639 adults with and without central obesity. Additionally, we employed a doubly robust estimation method to identify the association between central obesity and diabetes. Subjects with central obesity were 92% more likely to develop DM (HR = 1.65, 95%CI 1.12, 2.41). After adjusting for covariates, subjects with central obesity had a 72% increased risk of developing DM compared with subjects with non-central obesity in the PSM cohort (HR = 1.72, 95% CI 1.16, 2.56). Central obesity individuals had a 91% higher risk of DM than non-central obesity individuals, after adjustment for propensity score (HR = 1.91, 95% CI 1.29, 2.81). In sensitivity analysis, the central obesity group had a 44% (HR = 1.44, 95% CI 1.09, 1.90) and 59% (HR = 1.59, 95% CI1.35, 1.88) higher risk of DM than the non-central obesity group in the original and weighted cohorts after adjusting for confounding variables, respectively. Central obesity was independently associated with an increased risk of developing diabetes. After adjustment for confounding covariates, central obesity participants had a 72% higher risk of development of diabetes than non-central obesity individuals in the PSM cohort.

## Introduction

Diabetes Mellitus (DM) is a metabolic disorder with chronic hyperglycemia^1^. According to the epidemiological survey data released by the International Diabetes Federation in 2019, approximately 463 million adults between the ages of 20 to 79 years were diagnosed with diabetes globally, with an incidence of 9.3%^2^. DM is proving to be a global public health burden. It is estimated that global diabetes-related medical expenditure will be at least 2.1 trillion US dollars by 2030^3^. The complications of diabetes seriously affect people's health, such as diabetic retinopathy, cardiovascular accidents, and diabetic nephropathy^4–6^. Given the economic burden and health hazards brought by diabetes, the screening of high-risk populations for diabetes should be emphasized for early intervention. Therefore, it is vital to have some available and straightforward indicators to assess the risk of diabetes.

Adipose tissue is not only an energy storage area, but also acts as an endocrine and immune organ. Excessive accumulation of adipose tissue could affect metabolic function^7^. Obesity has been considered a risk factor for DM^8,9^. Central obesity is one type of obesity, and many central obesity indices have been established, including waist-to-height ratio, waist circumference, waist-to-hip ratio, and so on^10^. At present, central obesity was positively associated with the risk of diabetes in most pre-existing studies^8,11–14^. However, most of these studies were cross-sectional or case–control studies, and the wide range of the hazard ratios/odds ratio for the association between central obesity and diabetes fluctuated from 1.33 to 11.83^8,14^. Besides, Mainous AG 3rd et al. and Sakashita Y et al. found that central obesity was not a risk factor for prediabetes and diabetes (OR:1.04, 95%CI:0.65–1.66 and HR:0.85, 95%CI:0.45–1.60)^15,16^. Current researches on the relationship between central obesity and DM are still controversial, and the range of hazard ratios/odds ratio also needs further precision. Therefore, the association between central obesity and DM still needs further study.

The previous studies mainly relied on traditional regression models. On the one hand, the traditional logistic regression model contains a relatively large number of variables and directly fits the data, so there is a problem of overfitting. On the other hand, prediction conclusions from traditional logistic regression models are unstable due to low event (diabetes) rates, and estimates of predictive power are overly optimistic^17,18^. Research methods based on propensity score (PS) are considered the core alternative for controlling the confounding of observational research. Both large and small sample theories show that adjustment for the scalar PS is sufficient to remove bias due to all observed covariates^19,20^. Several adjustment methods incorporating the estimated PS have been proposed, including propensity score matching, propensity score matching, propensity score adjustment, propensity score weighting, propensity score stratified analysis, and we could scientific analysis of central obesity and diabetes relationship. Furthermore, using these methods, we could quantify the relationship between central obesity and diabetes and give a range of hazard ratios. Therefore, we can derive a more precise range of the relationship between central obesity and diabetes through this analysis using different methods in the Japanese NAGALA (NAfld in Gifu region, longitudinal analysis) database.

## Methods

### Study design

The present retrospective study was conducted using 2004 and 2015 records from NAGALA (NAfld in the Gifu Area, Longitudinal Analysis) database provided by Murakami Memorial Hospital in Japan. The interesting independent variable in the present work was central obesity. The dependent variable was DM.

### Data source

The data for our study came from the Dryad Digital Repository (https://datadryad.org/), which allows users to download data for free. Our data was provided by Takuro Okamura et al. from Ectopic fat obesity presents the greatest risk for incident type 2 diabetes: a population-based longitudinal study. Dryad, Dataset, https://datadryad.org/stash/dataset/doi:10.5061%2Fdryad.8q0p192^21^. Under the premise of not infringing on the rights of the author, users can use the site's data for data analysis free of charge based on Dryad Terms of Service. All study methods are conducted following relevant regulations and guidelines, and a statement is included in the Declarations section.

### Study participants

The original study investigated the effect of obesity phenotypes on the risk of incident diabetes using the NAGALA database. The center, where the programs were performed, was founded in 1994, evaluates > 8000 medical exams annually and 60% of participants receive one to two exams per year. Since many participants have undergone repeated examinations, all included in the original study were subjected to repeated inspections. The original data were obtained from NAGALA database provided by Murakami Memorial Hospital in Japan. We got the details of the NAGALA study from the original article, which recruited 20,944 participants who took the physical exam between 2004 and 2015 and completed at least a second exam.

**Figure 1:**
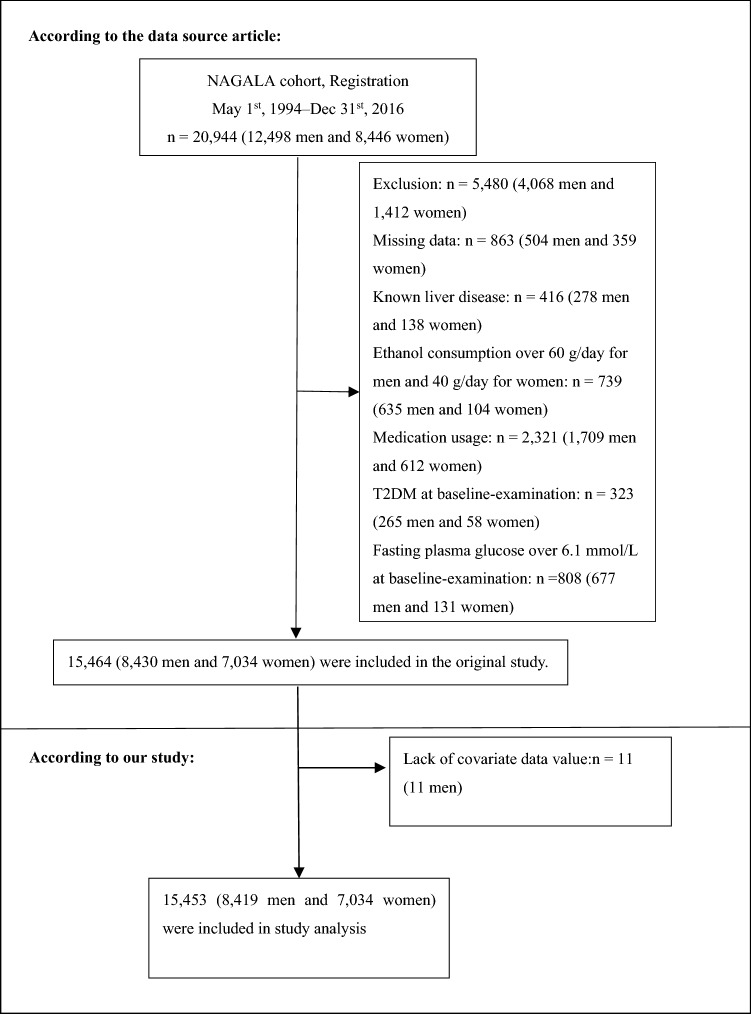
Study population.

The original study initially enrolled 20,944 Japanese individuals who took the physical exam between 2004 and 2015 and completed at least a second exam. Individuals were excluded if they met any of the following criteria: (1) alcoholic fatty liver disease; (2) viral hepatitis (detection of hepatitis B antigen and hepatitis C antibody at baseline) ; (3) using any medication at baseline; (4) diabetes at baseline; (5) missing data of covariates; (6) fasting plasma glucose (FPG) ≥ 6.1 mmol/L. In this study, we further excluded participants with a lack of covariate data (11 had no high-density lipoprotein cholesterol values). Finally, 5491 subjects were excluded, and left 15,453 subjects were left for data analysis this study (see detailed flowchart in Fig. 1).

### Definition of central obesity

At baseline, the independent variable was central obesity, defined as a waist-to-height ratio ≥ 0.5 as a cut-off^22,23^.

### Diagnosis of incident diabetes

Our interesting outcome variable was DM. DM was defined as participants whose HbA1c was not lower than 6.5%, FPG was not lower than 7 mmol/l, or self-reported during follow-up^24^.

### Covariates

Covariates were selected in our study according to our clinical experience and the previous literature. Therefore, the present study selected the following variables as covariates according to the above principles: (1) continuous variables: age, body mass index (BMI), systolic blood pressure (SBP), diastolic blood pressure (DBP), alcohol consumption, gamma-glutamyl transferase (GGT), total cholesterol (TC), triglycerides (TG), alanine aminotransferase (ALT), aspartate aminotransferase (AST), glycosylated hemoglobin A1c (HbA1c), FPG, and high-density lipoprotein cholesterol (HDL-C) ; (2) categorical variables: gender, smoking status, regular exerciser. In the original research, data on all subjects' medical history and lifestyle factors were collected through a standardized self-management questionnaire. Clinical measurements of height, WC, weight, and blood pressure were performed by professional staff. The original study staff followed a uniform procedure to obtain laboratory test results under standardized conditions. We divided the subjects into three groups based on alcohol consumption: moderate alcohol consumption (140–210 g/week, light alcohol consumption (40–140 g/week), and no or very little alcohol consumption (< 40 g/week)^25^. The original study defined regular exercise as playing any type of exercise > 1 × /week^26^.

### Statistical analyses

Mean ± standard deviation (SD) (Gaussian distribution) or median (interquartile ranges) (Skewed distribution), were reported for continuous variables, and frequencies and percentages were presented for categorical variables. The wilcoxon rank-sum, the tests two-sample t-tests, and the χ^2^ test were presented to test for differences between the groups. We use the mean of HDL-C to handle missing values.

PS analysis matched the characteristics at baseline between the central obesity and non-central obesity groups (see detailed flowchart in Table 1) and formed a single group of subjects with similar characteristics at baseline. We calculated PS on the basis of central obesity as an independent variable and 16 variables at baseline as covariates using a non-reduced multivariate logistic regression model^27^. The current study was applied to the use of a 1:1 matching protocol without replacement (greedy-matching algorithm), with a caliper width equal to 0.01^28^. Standardized differences were an evaluation index of evaluating the balance between groups. A given covariate was considered a relatively small imbalance if the standardized difference is less than 10.0%^29^. In addition, we applied the Kaplan–Meier method to calculate the probability of DM-free survival in each group and confirmed significance by the log-rank test. The current study applied Cox proportional hazards regression model to examine the association between central obesity and DM incidence in the PSM cohort. The current study used a doubly robust estimation method combining PS model and multivariate regression model to test the relationship between central obesity and DM incidence^30^. The stratified binary logistic regression model was conducted to subgroup analysis in various subgroups (age, gender, BMI, ALT, AST, GGT, HbA1c, FPG, TC, TG, HDL-C, and PS). Firstly, we converted the continuous variable age(< 50, ≥ 50 years), ALT, AST, GGT, HbA1c, FPG, TC, TG, HDL-C, PS to a categorical variable based on the clinical cut point or median, or tertiles^31^. Secondly, in addition to the stratification factor itself, we adjusted for all factors (age, gender, BMI, ALT, AST, GGT, HbA1c, FPG, TC, TG, HDL-C and PS). Each stratification was adjusted for all factors, except for the stratification factor. In the subgroup analysis, only corresponding matched pairs in the same subgroup were selected to ensure a balance of characteristics at baseline between the central obesity and non-central obesity groups. For example, in the subgroup of participants under the age of 50, only when the matched pairs of the central obesity and non-central obesity groups both belonged to the subgroup under the age of 50, these participants could be included in the subgroup analysis. Likelihood ratio tests were used to inspect the modifications and interactions of the subgroups. Lastly, the current study employed likelihood ratio tests to examine the interactions and modifications of subgroups^32,33^.

**Table 1:** Baseline characteristics before and after propensity score matching.

Before matching				*P*	After matching			
Characteristic	Noncentral obesity	Central obesity	Standardized difference (100%)		Noncentral obesity	Central obesity	Standardized difference (100%)	*P*
Participants	12,092	3361			1639	1639		
Age (years)	42.85 ± 8.65	46.82 ± 9.07	44.9	< 0.001	46.82 ± 8.62	46.94 ± 9.05	1.3	0.701
BMI (kg/m^2^)	21.06 ± 2.26	25.92 ± 2.84	189.2		23.98 ± 1.67	24.05 ± 1.68	4.4	0.326
Waist-to-height ratio	0.45 ± 0.04	0.51 ± 0.05	133	< 0.001	0.48 ± 0.02	0.52 ± 0.02	229	< 0.001
**Gender**			23.5	< 0.001				1.000
Male	6285 (51.98%)	2134 (63.49%)			1008 (61.50%)	1008 (61.50%		
Female	5807 (48.02%)	1227 (36.51%)			631 (38.50%)	631 (38.50%)		
SBP (mmHg)	112.06 ± 13.89	123.25 ± 15.44	76.2	< 0.001	119.33 ± 14.30	119.33 ± 14.93	< 0.1	0.991
DBP (mmHg)	69.91 ± 9.85	77.59 ± 10.59	75.1	< 0.001	75.07 ± 10.15	75.03 ± 10.36	0.4	0.910
FPG (mg/dL)	92.13 ± 7.37	95.98 ± 6.89	54.0	< 0.001	94.84 ± 7.24	94.91 ± 6.95	1.0	0.785
HbA1c (%)	5.14 ± 0.31	5.27 ± 0.34	40.6	< 0.001	5.21 ± 0.33	5.22 ± 0.33	2.0	0.561
ALT (U/L)	16 (12, 21)	22 (16, 32)	58.9	< 0.001	19 (14, 26)	20 (15, 28)	0.6	0.857
AST (U/L)	17 (14, 20)	19 (16, 24)	39.2	< 0.001	18 (14, 22)	18 (15, 22)	0.7	0.843
GGT (U/L)	14 (11, 20)	21 (14, 31)	43.4	< 0.001	17 (12, 27)	19 (13, 28)	1.1	0.762
TC (mg/dL)	194.93 ± 32.57	210.04 ± 33.71	45.6	< 0.001	205.22 ± 33.61	205.45 ± 33.62	0.7	0.850
TG (mg/dL)	59 (41,88)	95 (63, 139)	64.0	< 0.001	84 (58, 123)	84 (55, 125)	2.1	0.554
HDL-C (mg/dL)	58.56 ± 15.62	49.29 ± 13.01	64.5	< 0.001	51.64 ± 13.62	51.45 ± 14.00	1.3	0.707
Ethanol consumption (g/week)	1 (0, 60)	2.80 (0, 84)	8.8	< 0.001	2.8 (0, 84)	2.8 (0,87.5)	0.7	0.852
**Smoking status**			15.8	< 0.001			2.0	0.851
Never smoker	7264 (60.07%)	1763 (52.45%)			882 (53.81%)	877 (53.51%)		
Ever smoker	2185 (18.07%)	764 (22.73%)			386 (23.55%)	378 (23.06%)		
Current smoker	2643 (21.86%)	834 (24.81%)			371 (22.64%)	384 (23.43%)		
**Regular exerciser**			10.1	< 0.001			0.7	0.850
No	9876 (81.67%)	2871 (85.42%)			1369 (83.53%)	1373 (83.77%)		
Yes	2216 (18.33%)	490 (14.58%)			270 (16.47%)	266 (16.23%)		

Values were n (%) or mean ± SD or median (interquartile range: 25th to 75th percentiles).

*SD* standard deviation, *BMI* body mass index, *SBP* systolic blood pressure, *DBP* diastolic blood pressure, *FPG* fasting plasma glucose, *HbA1c* glycosylated haemoglobin, *ALT* alanine aminotransferase, *AST* aspartate aminotransferase, *GGT* gamma-glutamyl transferase, *TC* total cholesterol, *TG* triglyceride, *HDL-C* high-density lipoprotein cholesterol.

For sensitivity analyses, the estimated PS was used to calculate the inverse probability of treatment weights (IPTW). For example, IPTW was calculated as 1/PS for subjects with central obesity and 1/(1-PS) for subjects with non-central obesity. IPTW model applied to create weighted cohort^34^. The current study used a series of sensitivity analyses to examine the robustness of the findings and how applying different models of associative inference affects the conclusions. Two relationship inference models were employed to the original and weighted cohorts in the sensitivity analysis. The calculated p-values and effect sizes were reported and compared in all models. Based on the STROBE statement^35^, the results of the current study were reported.

All analyses in our study were performed with the Empower-Stats (http://www.empowerstats.com, X&Y Solutions, Inc., Boston, MA) and the statistical package R (http://www.R-project.org, R foundation). P < 0.05 (two-sided) was considered statistically significant.

### Ethics approval and consent to participate

This study was conducted under the approval of the institutional review board of the Murakami Memorial Hospital, and was conducted in accordance with the ethical principles of the Declaration of Helsinki. Informed consent was obtained from all patients. The original researchers encoded their identity information as non-traceable codes to ensure participants' privacy and data anonymization.

## Results

### Characteristics of participants

In this study, 15,453 subjects were finally included, of whom 54.48% were male. Among them, 3361(21.75%) participants suffered from central obesity. The mean age was 43.71 ± 8.90 years in the study. During a mean follow-up of 6.05 ± 3.78 years, 373 subjects developed diabetes. Before PSM, all baseline characteristics in Table1 exhibited statistically significant differences between the central and non-central obesity groups. Table 1 showed that subjects with central obesity were older, had a higher BMI, had higher blood pressure, and normally had higher levels of AST, ALT, GGT, TC, TG, FPG, and HbA1c. The higher percentage of males, ethanol consumption, ever smoker and current smoker were observed in the central obesity group. In addition, subjects with central obesity had a lower HDL-C level and a lower rate of regular exercise than subjects with non-central obesity. By using a 1:1 PSM, 1639 central obesity subjects were finally matched with 1639 non-central obesity subjects. After PSM, the standardized differences for all covariates were < 10.0%, indicating a good match. That is, the differences were minimal in characteristics at baseline between the two groups.

### Incidence rate of diabetes

Table 2 showed the incidence of diabetes by central obesity exposure before and after propensity-score matching. Before propensity-score matching, a total of 373 participants developed incident diabetes during follow-up. The morbidity rate in the overall population was 399.137 per 100,000 person-years, specifically, 1097.319 per 100,000 person-years in the central obesity group and 215.011 per 100,000 person-years in the non-central obesity group, respectively. The corresponding cumulative incidence of diabetes in the central obesity and non-central obesity group were 6.367(5.541–7.193) and 1.315(1.112–1.518), respectively. This crude difference in the morbidity rate between the two groups changed significantly after the PS-matching procedure (563.728 per 100,000 person-years among the overall population, 739.772 per 100,000 person-years among the central obesity subjects, and 391.660 per 100,000 person-years among the non-central obesity subjects). The corresponding cumulative incidence in the central obesity and non-central obesity group were 4.393 (3.400–5.386) and 2.380 (1.641–3.118), respectively.

**Table 2:** Incidence rate of incident diabetes before and after propensity-score matching.

Variable	Participants (n)	DM events (n)	Cumulative incidence (95% CI)	Per 100,000 person-year
**Before matching**				
Total	15,453	373	2.414 (2.172–2.656)	399.137
Central Obesity	3361	214	6.367 (5.541–7.193)	1097.319
Noncentral obesity	12,092	159	1.315 (1.112–1.518)	215.011
**After matching**				
Total	3278	111	3.386 (2.767–4.006)	563.728
Central Obesity	1639	72	4.393 (3.400–5.386)	739.772
Noncentral obesity	1639	39	2.380 (1.641–3.118)	391.660

*CI* confidence interval, *DM* diabetes mellitus.

**Figure 2:**
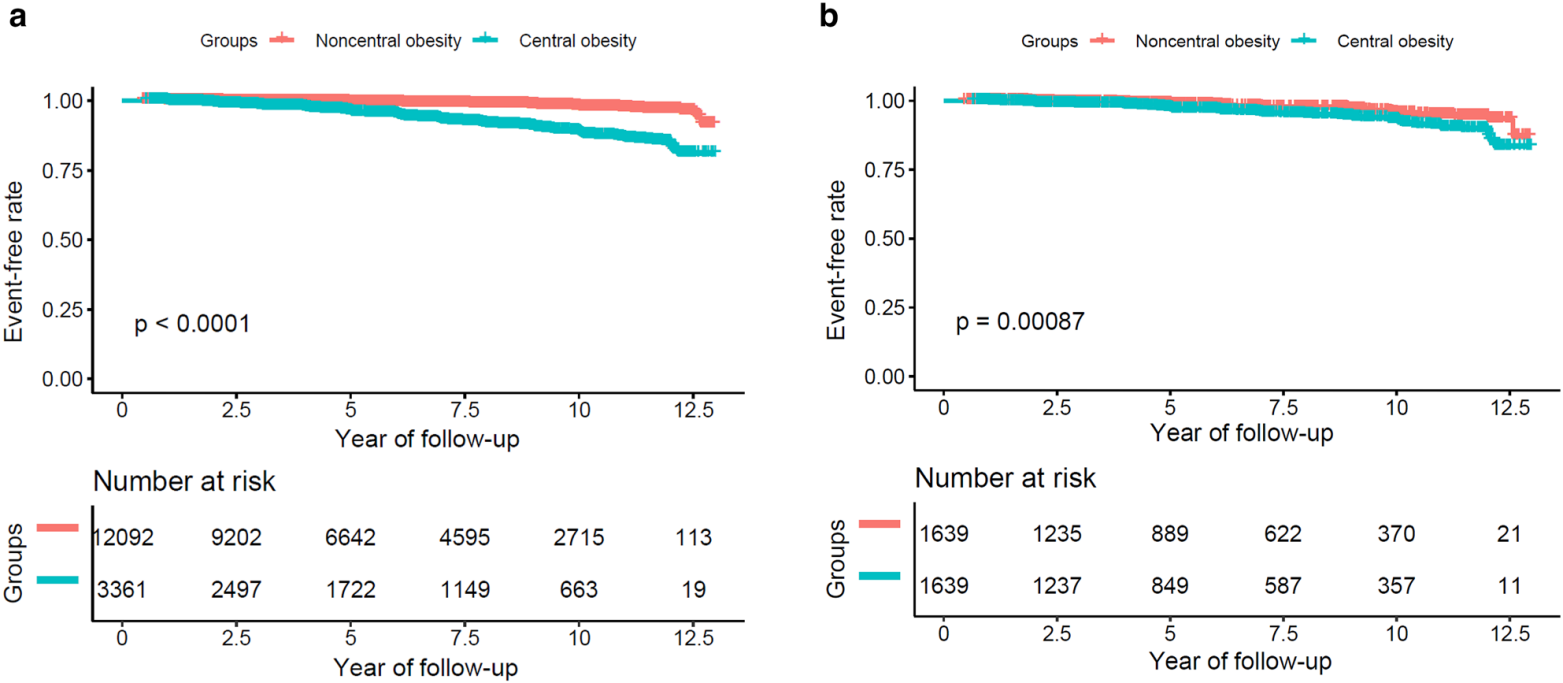
(**a**) Kaplan–Meier analysis of incident diabetes based on Central obesity and Noncentral obesity in the original cohort (log-rank, P < 0.0001). (**b**) Kaplan–Meier analysis of incident diabetes based on Central obesity and Noncentral obesity in the propensity score matching cohort (log-rank, P = 0.00087).

The Kaplan–Meier analysis results showed that the probability of DM-free survival among the subjects with central obesity was obviously lower than that of those with non-central obesity before PSM (P < 0.0001; Fig. 2a). After PSM, this difference remained unchanged (P = 0.00087; Fig. 2b).

### The results of association between central obesity and incident DM

This current study applied cox proportional hazard regression model to evaluate the associations between central obesity and incident diabetes in the PSM cohort. This current study simultaneously presented the unadjusted, minimally adjusted, fully adjusted, and PS adjusted models in Table 3. In the unadjusted model, central obesity was a significant association with the risk of diabetes. Subjects with central obesity were 92% more likely to develop DM (HR = 1.92, 95% CI 1.30–2.83, P = 0.0011). After adjusting the partial confounding covariates (adjusted for age, BMI, gender, smoking status, ethanol consumption, regular exerciser, SBP, DBP), the association between central obesity and incident diabetes still did not change significantly (HR = 1.78, 95% CI 1.20–2.64, P = 0.0039). In the fully adjusted model (adjusted for age, BMI, gender, smoking status, ethanol consumption, regular exerciser, SBP, DBP, ALT, AST, GGT, HbA1c, FPG, TC, TG, HDL-C), the association between central obesity and incident diabetes was still detected (HR = 1.72, 95% CI 1.16–2.56, P = 0.0074). Subjects with central obesity had a 72% increased risk of developing diabetes compared with subjects with non-central obesity. After adjusting for PS, the association was still detected, and participants with central obesity had a 91% higher risk of diabetes (HR = 1.91, 95% CI 1.29–2.81, P = 0.0012).

**Table 3:** Association between central obesity and incident diabetes in different models.

Variable	Non-adjusted (HR, 95% CI, *P*)	Model I (HR, 95% CI, *P*)	Model II (HR, 95% CI, *P*)	Model III (HR, 95% CI, *P*)
Non-central obesity	Ref	Ref	Ref	Ref
Central obesity	1.92 (1.30, 2.83), 0.0011	1.78 (1.20, 2.64), 0.0039	1.72 (1.16, 2.56), 0.0074	1.91 (1.29, 2.81), 0.0012

Crude model: we did not adjust for other covariates.

Model I: we adjusted for age, BMI, gender, smoking status, ethanol consumption, regular exerciser, SBP, DBP.

Model II: we adjusted for age, BMI, gender, smoking status, ethanol consumption, regular exerciser, SBP, DBP, ALT, AST, GGT, HbA1c, FPG, TC, TG, HDL-C.

Model III: we adjusted for propensity score.

*HR* hazard ratios, *CI* confidence interval, *Ref* reference.

### The results of subgroup analysis

The current study applied subgroup analysis to consider other influencing variables, such as gender, that might have influenced the results on the relationship between central obesity and the risk of diabetes. The current study chosen gender, BMI, age, FPG, HbA1c, TC, TG, HDL-C, ALT, AST, GGT, and PS as the stratification variables to detect the trend of effect sizes in these variables (Table 4). The results showed that none of the interactions were observed based on the prior specifications. On the basis of the previous norm, our study did not observe any of the interactions. After PSM, the finding showed that the above variables did not modify the relationship between central obesity and diabetes risk.

**Table 4:** Effect size of central obesity on incident diabetes in prespecified and exploratory subgroups.

Characteristic	No of participants	HR (95%CI)	*P* value	P for interaction
**Gender**				0.9171
Male	1240	2.12 (1.09, 4.13)	0.0269	
Female	486	3.15 (0.27, 36.24)	0.3568	
**BMI**				0.7981
< 25 kg/m^2^	1946	1.59 (0.89, 2.84)	0.1202	
≥ 25 kg/m^2^	502	1.44 (0.61, 3.39)	0.4017	
**Age**				0.3739
< 50	1238	1.68 (0.84, 3.37)	0.1453	
≥ 50	518	0.87 (0.25, 2.95)	0.8181	
**FPG (mg/dL)**				0.9958
Low	802	1.02 (0.06, 16.79)	0.9882	
High	874	1.77 (0.91, 3.43)	0.0911	
**HbA1c (%)**				0.4800
Low	634	0.88 (0.22, 3.53)	0.8577	
High	1046	1.61 (0.88, 2.93)	0.1216	
**TC (mg/dL)**				0.6490
Low	818	1.57 (0.56, 4.46)	0.3927	
High	840	1.23 (0.50, 3.05)	0.6530	
**TG (mg/dL)**				0.3787
Low	798	2.89 (0.70, 12.02)	0.1441	
High	828	1.54 (0.77, 3.07)	0.2241	
**HDL-C (mg/dL)**				0.3523
Low	804	1.41 (0.70, 2.84)	0.3359	
High	818	0.68 (0.18, 2.56)	0.5665	
**ALT (U/L)**				0.5674
Low	700	2.77 (0.59, 12.90)	0.1955	
High	964	1.81 (0.96, 3.42)	0.0678	
**AST (U/L)**				0.9342
Low	676	1.07 (0.22, 5.13)	0.9330	
High	1012	1.20 (0.60, 2.40)	0.5972	
**GGT (U/L)**				0.1643
Low	776	0.82 (0.16, 4.31)	0.8126	
High	896	3.10 (1.35, 7.12)	0.0076	

The above model has been adjusted for age, BMI, gender, smoking status, ethanol consumption, regular exerciser, SBP, DBP, ALT, AST, GGT, HbA1c, FPG, TC, TG, HDL-C.

In each case, the model was not adjusted for the stratification variable.

### Sensitivity analysis

In order to test the robustness of our findings, our study used a series of sensitivity analysis to evaluate the association between central obesity and diabetes risk in both the weighted cohort and original cohort. Our study created a weighted cohort by inverse probability of treatment weights. In addition, Table 5 provided the unadjusted, partially adjusted, and fully adjusted models in both cohorts. Our findings revealed that central obesity was strongly associated with incident diabetes in these two cohorts. The central obesity group had a 44% (HR = 1.44, 95% CI 1.09–1.90, P = 0.0096) and 59% (HR = 1.59, 95% CI 1.35–1.88 P < 0.0001) higher risk of DM than the non-central obesity group in the original and weighted cohorts after adjusting for confounding variables, respectively.

**Table 5:** Association between central obesity and incident diabetes in different models of the original and the weighted cohort.

Variable	Non-adjusted	Model I (HR,95%CI, *P*)	Model II (HR,95%CI, *P*)
**(A)**			
Non-central obesity	Ref	Ref	Ref
Central obesity	5.25 (4.28, 6.45), < 0.0001	1.82 (1.38, 2.40), < 0.0001	1.44 (1.09, 1.90), 0.0096
**(B)**			
Non-central obesity	Ref	Ref	Ref
Central obesity	2.43 (2.09, 2.84), < 0.0001	1.65 (1.41, 1.94), < 0.0001	1.59 (1.35, 1.88), < 0.0001

(A) In the original cohort; (B) in the weighted cohort.

Crude model: we did not adjust for other covariates.

Model I: we adjusted for age, BMI, gender, smoking status, ethanol consumption, regular exerciser, SBP, DBP.

Model II: we adjusted for age, BMI, gender, smoking status, ethanol consumption, regular exerciser, SBP, DBP, ALT, AST, GGT, HbA1c, FPG, TC, TG, HDL-C.

*HR* hazard ratios, *CI* confidence interval, *Ref* reference.

## Discussion

The PSM cohort study revealed that central obesity was an independent risk factor for the development of diabetes after adjusting for the confounding factors. The diabetes risk increased by 72% in the subjects with central obesity. After adjusting for the PS, the diabetes risk decreased to 91%. In subgroup analysis, we observed no interaction, suggesting that our results were robust. The association between central obesity and diabetes could also be detected in the weighted and original cohorts.

Central obesity was strongly associated with cardiovascular disease, diabetes, dyslipidemia, and hypertension, even in lean or simply overweight patients by body mass index^36,37^. Compared with the general obesity indicator, abdominal obesity indicators strongly correlate with prediabetes^38^. The analysis compared the quantitative results of all available epidemiological studies and revealed that central obesity significantly increased the risk of type 2 diabetes across a range of different ethnic groups^39^. The improvement of central obesity could reduce the chance of developing DM^40,41^. A large cross-sectional study of 42,116 older adults from several countries found that central obesity was associated with significantly higher odds for DM after adjusting for various confounding variables, which was the same as our findings^42^. However, the diagnosis of DM was based on self-reported diagnosis in that study, and since it was a cross-sectional study, temporal association or causality could not be concluded. Therefore, the findings of these studies could not be applied to the general population. In our study, in addition to self-reported during follow-up, diabetes mellitus was also defined as FPG ≥ 7 mmol/L, HbA1c ≥ 6.5%. Besides, our study was a retrospective cohort study which reduces the risk of selection and observational bias. Our findings could better reveal the true link between central obesity and diabetes. In contrast, there were also some studies that have shown inconsistent conclusions. After adjusting for confounding covariate, the relationship between central obesity and the DM risk was insignificant^15,43,44^. We analyzed possible reasons for these inconsistent results as follows: (1) Different researches had included diverse populations, such as different ages, different genders, and different races. (2) The sample size varied widely among researchers. (3) Different researches adjusted for different confounding covariates that influenced the association between central obesity and DM. (4) The length of follow-up varied greatly, which affected the incidence of diabetes. (5) Our study excluded participants with a heavy drinking habit, viral hepatitis, FPG ≥ 6.1 mmol//L, or any medication usage at baseline.

Athletes with increased body mass may be incorrectly classified as obese, while people with low lean but high body fat may still have a normal BMI^45^. In contrast, surrogate measures of the waist-to-height ratio anthropometric measurements were used to assess central fat distribution^46^. Previous studies have shown that female sex, smoking, lack of exercise, hyperlipidemia, and GGT are closely associated with central obesity^47–49^. Our study shows that the prevalence of central obesity is 21.7%. One in five participants had central obesity. This study found that females, the elderly, lack of exercise, high BMI, TC, GGT, HbA1c, and low HDL-C were independent risk factors for central obesity. Our study suggests that waist circumference and height measurement should be recommended as a simple and effective tool for screening diabetes risk. These individuals often require appropriate health education and timely intervention to manage and/or prevent the development of diabetes. At the same time, it also provides a new perspective for preventing diabetes: under the same conditions, even if the BMI is the same, you should pay attention to your waist-to-height ratio. Therefore, by controlling for these factors, we can reduce the incidence of central obesity and thus curb the growing prevalence of central obesity, which can significantly affect the already overburdened health care system.

In our study, the doubly robust estimation method presented a significant relationship between central obesity and incident diabetes in the PSM cohort. Central obesity raised the risk of developing DM by 72%. And after adjusting PS, the figure dropped to 91%. The HR for DM in our study (HR = 1.72) was relatively lower than in previous research (HR = 2.5)^50^. The incidence of diabetes in our study was 3.99 per 1,000 person-years, compared with 8.8 per 1,000 person-years in Japanese during the same period^51^. The incidence of diabetes in our study was significantly lower than the incidence of diabetes in Japanese during the same period. The difference might be that we applied a PSM method to minimize the effect of potentially confounding covariates, so our findings better represent the real-world association between central obesity and DM. In addition, the confounding factors we adjusted were different. We adjusted for more clinical and laboratory parameters, including exercise, ALT, AST, GGT, HbA1c, FPG, TC, TG and HDL-C, and so on. Evidence showed that those parameters were associated with central obesity and incident diabetes^52–54^. In addition, on the basis of a large sample (15,453 subjects), our study had strengthened statistical power. The findings of our study provided support for an adverse effect of central obesity on the development of DM. A detailed understanding of central obesity as a potential risk factor for DM will assist us in better understanding and communicating risks with patients and make more individualized management and prevention regimens. In the past, PSM analysis was mainly used to compare different treatments. Our study will help generalize the PSM method in related research.

Several hypotheses have been advanced to explain the relationship between abdominal fat accumulation and DM risk. The mechanisms are as follows: (1) Hypertrophied intra-abdominal adipocytes are characterized by a hyperlipolytic state that is resistant to the antilipolytic effect of insulin. The resulting non-esterified fatty acid flux to the liver may impair liver metabolism, leading to increased hepatic glucose production^55^. (2) Some studies showed that excess visceral fat is associated with increased insulin resistance and high levels of inflammatory cytokines (TNF-alpha and hs-CRP), and decreased levels of adiponectin^56^. (3) Abdominal fat distribution was considered as the driver of metabolic complications. It remains to be determined whether metabolic complications arise due to the accumulation of ceramide or other lipids, such as diacylglycerol, associated with mitochondrial dysfunction^57^.

### Study strengths and limitations

Our study has some strengths, and we listed them as follows. Our study has some strengths, and we listed them as follows. First, to our knowledge, our study is the first to use the PSM method to test the association between central obesity and DM risk. The PSM method has many advantages, including achieving the effect of “similar randomization”, balancing inter-group confounders, and minimizing of inter-group differences. Second, we performed subgroup analysis to reveal other potential risk factors influencing the relationship between central obesity and diabetes. Third, this study performed a series of sensitivity analysis to confirm the robustness of the results. The current study used IPTW to create a weighted cohort and further tested the relationship between central obesity and DM incidence in the weighted cohort. Fourth, compared to most previous retrospective studies, the sample size of our study was larger.

This research has the following shortcomings and needs attention. First, the subjects were all of the Japanese ancestries in this study. Therefore, studies in other ethnic groups are required to confirm our results' generalizability further. Second, the diabetes incidence in our study might have been underestimated due to the lack of a 2-h oral glucose tolerance test. However, conducting a 2-h oral glucose tolerance test is not feasible in such a large cohort. Third, the PSM method can confirm the balance of the covariates that have been measured. Still, it does not verify the balance of unmeasured confounding covariates. In order to minimize the interference of variables on the results, we set the caliper width to 0.01. Fourth, when the weights for a small number of subjects are extremely large, weighted methods are likely to perform poorly. Even though some approximate fixes have been described, no perfect solution has yet been found^58^. These few large weights mean that, without making additional a priori assumptions, it is impossible to obtain accurate estimates of the population parameters through the weighted method. The estimated standard-error-of-treatment effect, in this case, might underestimate the actual measure between the weighted estimator and the estimate of the population parameter. We conducted a set of sensitivity analyses to ensure the reliability of the results. Fifth, propensity score methods are only aimed at reducing bias. For example, suppose BMI is a risk factor strongly associated with the outcome but independent of central obesity. In that case, one may want to adjust for a BMI in addition to the propensity score adjustment to improve efficiency, as failing to control for BMI may not cause bias but may still result in a poor estimate. Sixth, the differences between type 1 diabetes, type 2 diabetes mellitus, and gestational diabetes were not considered in the present study. However, type 2 diabetes mellitus is most common, accounting for over 90% of the cases of diabetes mellitus. Therefore, this study explored the association between central obesity and type 2 diabetes mellitus. Seventh, the incident outcomes (e.g., competing risk of death) were not censored. This is a secondary retrospective study, and the data was downloaded from a computerized database established by the Murakami Memorial Hospital in Japan. The raw data did not provide other incident outcomes. And the raw data only provided DM incidents. Seventh, our study did not exclude patients with undiagnosed diabetes. We excluded participants with baseline-diagnosed diabetes and FPG ≥ 6.1 mmol/L. Eighth, in the real world, there are more people with non-central obesity than people with central obesity. We did not perform 1-to-m propensity score matching due to data limitations. Finally, the incidence of diabetes in our study was 3.99 per 1000 person-years, compared with 8.8 per 1000 person-years in Japanese during the same period^51^. The incidence of diabetes in our study was significantly lower than the incidence of diabetes in Japanese during the same period, which may be related to our exclusion of participants with heavy drinking habits, viral hepatitis, FPG ≥ 6.1 mmol/L, or any drug use at baseline.

## Conclusions

Central obesity was an independent risk factor for the development of DM. After adjusting for the confounding factors, the risk of developing diabetes in the central obesity participants increased by 72% compared with non-central obesity subjects in the PSM cohort. The subjects with central obesity had a 91% increased risk of DM after adjusting for PS. Therefore, this study provides clinical reference evidence for preventing diabetes risk by controlling central obesity (supplementary information).

## Supplementary Information

Supplementary Tables.

## Data Availability

The datasets generated and/or analysed during the current study are available in the ‘DataDryad’ repository, (https://datadryad.org/stash/dataset/doi:10.5061%2Fdryad.8q0p192).

## Abbreviations

BMI: Body mass index
SBP: Systolic blood pressure
DBP: Diastolic blood pressure
FPG: Fasting plasma glucose
HbA1c: Glycosylated hemoglobin A1c
ALT: Alanine aminotransferase
AST: Aspartate aminotransferase
GGT: Gamma-glutamyl transferase
TC: Total cholesterol
TG: Triglyceride
HDL-C: High-density lipoprotein cholesterol
DM: Diabetes mellitus
SD: Standard deviation
HR: Hazard ratios
CI: Confidence intervals
Ref: Reference
PS: Propensity score
PSM: Propensity score matching
IPTW: Inverse probability of treatment weights
