# Supplementary material for: Association between central obesity and incident diabetes mellitus among Japanese: a retrospective cohort study using propensity score matching
Source: Sci Rep. 2022 Aug 4;12:13445. doi: 10.1038/s41598-022-17837-1 (PMC9352654; doi:10.1038/s41598-022-17837-1)
Supplement: Supplementary file 1 — Supplementary Tables. [file 41598_2022_17837_MOESM1_ESM.docx]

**Table 1S The results of Univariable and Multivariable analysis**

|  | Univariable (OR,95% CI, P) | Multivariable (OR,95% CI, P) |
| --- | --- | --- |
| Gender |  |  |
| Women | ref | ref |
| Men | 1.61 (1.49, 1.74) <0.0001 | 0.26 (0.22, 0.31) <0.0001 |
| Age(years) | 1.05 (1.05, 1.06) <0.0001 | 1.09 (1.08, 1.10) <0.0001 |
| Ethanol consumption(g/week) | 1.00 (1.00, 1.00) <0.0001 | 1.00 (1.00, 1.00) 0.3243 |
| Smoking status |  |  |
| Never-smoker | ref | ref |
| Ex-smoker | 1.44 (1.31, 1.59) <0.0001 | 0.97 (0.82, 1.14) 0.6733 |
| Current-smoker | 1.30 (1.18, 1.43) <0.0001 | 0.89 (0.75, 1.05) 0.1679 |
| Regular exerciser |  |  |
| No | ref | ref |
| Yes | 0.76 (0.68, 0.85) <0.0001 | 0.66 (0.56, 0.77) <0.0001 |
| SBP(mmHg) | 1.05 (1.05, 1.06) <0.0001 | 1.00 (0.99, 1.01) 0.6678 |
| DBP(mmHg) | 1.07 (1.07, 1.08) <0.0001 | 1.00 (0.99, 1.01) 0.9530 |
| BMI(kg/m^2^) | 2.36 (2.29, 2.43) <0.0001 | 2.67 (2.57, 2.78) <0.0001 |
| ALT (IU/L) | 1.05 (1.05, 1.06) <0.0001 | 1.01 (1.00, 1.01) 0.1095 |
| AST (IU/L) | 1.06 (1.05, 1.07) <0.0001 | 1.00 (0.99, 1.01) 0.8976 |
| GGT (IU/L) | 1.02 (1.02, 1.03) <0.0001 | 1.00 (1.00, 1.01) 0.0036 |
| HDL-C (mmol/L) | 0.95 (0.95, 0.96) <0.0001 | 0.99 (0.99, 1.00) 0.0010 |
| TG (mmol/L) | 1.01 (1.01, 1.01) <0.0001 | 1.00 (1.00, 1.00) 0.1760 |
| TC (mmol/L) | 1.01 (1.01, 1.01) <0.0001 | 1.00 (1.00, 1.01) 0.0013 |
| HbA1c (%) | 3.66 (3.24, 4.14) <0.0001 | 1.29 (1.06, 1.57) 0.0127 |
| FPG (mmol/L) | 1.08 (1.07, 1.08) <0.0001 | 0.99 (0.98, 1.00) 0.2478 |

**Table 2S Baseline characteristics before and after 1-to-2 propensity score matching.**

| Characteristic | Noncentral obesity | Central obesity | Standardized Difference (100%) | *P* |
| --- | --- | --- | --- | --- |
| **Participants** | 1666 | 833 |  |  |
| **Age（years）** | 45.78 ± 8.60 | 46.13 ± 8.95 | 4.1 | 0.3371 |
| **BMI (kg/m^2^)** | 23.05 ± 1.43 | 23.07 ± 1.37 | 2.0 | 0.6377 |
| **Gender** | 631 (37.9) | 351 (42.1) | 8.7 | 0.0441 |
| **Male** | 1035 (62.1) | 482 (57.9) |  |  |
| **Female** | 631 (38.50%) | 631 (38.50%) |  |  |
| **SBP (mmHg)** | 117.26 ± 13.89 | 116.55 ± 13.67 | 52 | 0.2243 |
| **DBP (mmHg)** | 73.75 ± 10.02 | 73.30 ± 9.74 | 4.6 | 0.2791 |
| **FPG (mg/dL)** | 94.12 ± 7.22 | 93.80 ± 6.94 | 4.5 | 0.2911 |
| **HbA1c (%)** | 5.18 ± 0.32 | 5.18 ± 0.32 | 1.8 | 0.6650 |
| **ALT(U/L)** | 20.87 ± 11.29 | 21.11 ± 11.37 | 2.1 | 0.6171 |
| **AST(U/L)** | 18.49 ± 6.22 | 18.79 ± 6.66 | 4.6 | 0.2726 |
| **GGT(U/L)** | 22.46 ± 19.58 | 22.77 ± 19.10 | 1.6 | 0.7092 |
| **TC (mg/dL)** | 202.27 ± 33.33 | 202.15 ± 32.51 | 0.3 | 0.9321 |
| **TG (mg/dL)** | 90.77 ± 59.77 | 89.09 ± 57.79 | 2.9 | 0.5039 |
| **HDL-C(mg/dL)** | 53.12 ± 14.72 | 53.42 ± 14.68 | 2.0 | 0.6315 |
| **Ethanol consumption (g/wk)** | 56.36 ± 88.40 | 54.59 ± 87.99 | 2.0 | 0.6357 |
| **Smoking status** |  |  | 3.6 | 0.7088 |
| **Never smoker** | 887 (53.2) | 458 (55) |  |  |
| **Ever smoker** | 389 (23.3) | 186 (22.3) |  |  |
| **Current smoker** | 390 (23.4) | 189 (22.7) |  |  |
| **Regular exerciser** |  |  | 4.9 | 0.2754 |
| **NO** | 1359 (81.6) | 695 (83.4) |  |  |
| **YES** | 307 (18.4) | 138 (16.6) |  |  |
